# A predictable prospect of the South Asian summer monsoon

**DOI:** 10.1038/s41467-022-34881-7

**Published:** 2022-11-18

**Authors:** Tuantuan Zhang, Xingwen Jiang, Song Yang, Junwen Chen, Zhenning Li

**Affiliations:** 1 School of Atmospheric Sciences, Sun Yat-sen University, Southern Laboratory of Ocean Science and Engineering (Zhuhai), Zhuhai, Guangdong 519082 China; 2 Guangdong Province Key Laboratory for Climate Change and Natural Disaster Studies, Sun Yat-sen University, Zhuhai, Guangdong 519082 China; 3 Heavy Rain and Drought-Flood Disasters in Plateau and Basin Key Laboratory of Sichuan Province, Institute of Plateau Meteorology, China Meteorological Administration, Chengdu, Sichuan 610072 China; 4 Shenzhen Wiselec Technology Co., Ltd., Shenzhen, Guangdong 518048 China; 5 Division of Environment and Sustainability, The Hong Kong University of Science and Technology, Hong Kong, China

**Keywords:** Atmospheric dynamics, Projection and prediction

## Abstract

Prediction of the South Asian summer monsoon (SASM) has remained a challenge for both scientific research and operational climate prediction for decades. By identifying two dominant modes of the SASM, here we show that the unsatisfactory prediction may be due to the fact that the existing SASM indices are mostly related to the less predictable second mode. The first mode, in fact, is highly predictable. It is physically linked to the variation of the Indian monsoon trough coupled with large rainfall anomalies over core monsoon zone and the northern Bay of Bengal. An index is constructed as a physical proxy of this first mode, which can be well predicted one season in advance, with an overall skill of 0.698 for 1979–2020. This result suggests a predictable prospect of the SASM, and we recommend the new index for real-time monitoring and prediction of the SASM.

## Abstract (web summary)

The authors identify a predictable component of the South Asian summer monsoon that is associated with the variability of the monsoon trough modulated by tropical sea surface temperatures.

## Introduction

The South Asian summer monsoon (SASM) exhibits significant variations in rainfall and atmospheric circulation on a wide range of time scales^1,2^. The interannual variability of SASM rainfall not only affects the lives of more than one billion people^3^, but also regulates local and remote atmospheric circulations by releasing diabatic heating^4–6^. Thus, substantial effort has been devoted to understanding the mechanisms and prediction of the SASM^4–8^. However, the operational seasonal prediction skill of the interannual variability of the SASM is quite low in recent decades, in terms of the popular All India Rainfall Index (AIRI)^8^. The AIRI is constructed based only on rainfall over the Indian subcontinent, ignoring the fact that a large amount of the SASM rainfall appears over the Bay of Bengal (BOB), which is the location of the maximum center of global atmospheric heat source^9^.

In addition to the AIRI, other indices have been proposed to quantify the interannual variability of the SASM based on rainfall and wind shear over South Asia. Dynamic indices are constructed by using the convection-circulation relationship in the tropics^10^ and the key features of the SASM, which include the lower-level westerly and upper-level easterly jet streams and local Hadley circulation^11,12^. One of the salient features of the SASM is the Indian monsoon trough (IMT), which extends from the northern BOB to western India^13^. The mesoscale convective systems embedded in the monsoon trough contribute a large proportion of rainfall to South Asia^14,15^. For example, most monsoon depressions, which account for almost all extreme rainfall events (rainfall >100 mm day^−1^) over central India, form over the warm waters of the northern BOB and move westward or northwestward along the IMT^16,17^. The dynamic and thermodynamic conditions differ over the eastern and western flanks of the IMT^18^. Nevertheless, the variation of the IMT over the BOB is not well considered by the existing monsoon indices.

The interannual variability of the SASM is forced by the sea-surface temperature (SST) anomalies related to El Niño-Southern Oscillation (ENSO), and their relationship has undergone apparent interdecadal changes^19,20^. The AIRI mostly represents the first leading mode of the Indian summer monsoon rainfall, which is concurrently associated with equatorial Pacific SST anomalies. The second mode is related to the IMT over northern India, which exhibits different rainfall anomalies and can be partially explained as a lagged response to ENSO^21^. It is not easy to predict the SASM successfully based on the AIRI due to the spring predictability barrier related to ENSO^11^. Nevertheless, because of the relatively persistent SST anomalies during the ENSO decaying phase^22^, the variability of the SASM related to the IMT may be better predicted compared to the AIRI.

This study is aimed at investigating the interannual variability of the SASM by considering the coupling variability between rainfall and atmospheric circulation over the entire South Asia rather than just the rainfall over the Indian subcontinent or large-scale circulation. Our focus is on the roles of convection and monsoon trough over the northern BOB in the interannual variability of the SASM. An index is proposed to represent the variability of the first mode, which reflects the tight coupled features of the SASM rainfall and low-level wind and is distinct from the anomalous patterns associated with the previous well-known monsoon indices. This monsoon index is closely related to tropical SST anomalies from the previous winter to the simultaneous summer, and can be well predicted one season ahead, exhibiting a predictable prospect of the SASM.

## Results

### Physical interpretations of the dominant SASM modes

**Fig. 1:**
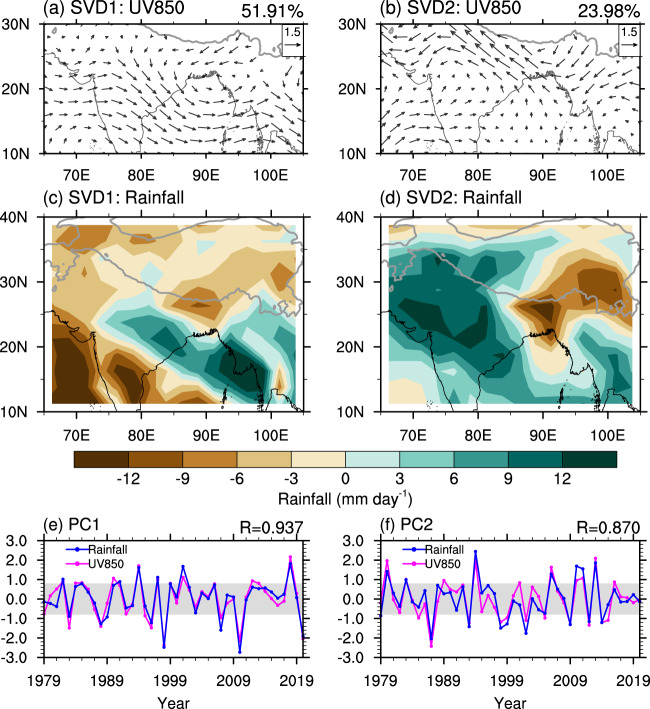
The first and second singular value decomposition modes of South Asian summer monsoon. Spatial patterns of **a**, **b** 850-hPa wind (m s^−1^; vector) and **c**, **d** rainfall (mm day^−1^; shading), and **e**, **f** corresponding standardized principal components (PCs) for the first **a**, **c**, **e** and second **b**, **d**, **f** singular value decomposition modes in summer (June-July-August). The explained squared covariance of each mode is presented at the top right of **a**, **b**. The magenta and blue curves in **e**, **f** represent the PCs for 850-hPa wind and rainfall, respectively, and their correlation coefficient is presented at the top right. Values within the gray zone (absolute values $\le$ 0.8) represent the normal years; and those below and above the gray zone are for the negative and positive years, respectively.

Singular value decomposition (SVD) analysis is performed based on normalized and detrended 850-hPa wind and rainfall over South Asia in June-August (JJA) during 1979–2020, to reflect the unique coupling characteristics between rainfall and low-level circulation of the SASM system. The SVD analysis is performed over the domain of (65°−105°E, 10°−30°N) for 850-hPa wind and the domain of (65°−105°E, 10°−40°N) for rainfall (see Fig. 1). We focus on the two leading modes that account for 75.89% of the total squared covariance fraction and are statistically distinguished from the rest of the eigenvectors according to the Monte Carlo approach. The first SVD (SVD1 hereafter) mode, accounting for 51.91% of the total squared covariance, is depicted by an anomalous low-level cyclonic circulation over the northern BOB and region south of the Tibetan Plateau (TP) (Fig. 1a) and by a nearly northwest-southeast elongated sandwich-like pattern of rainfall anomalies, with positive anomalies along the central India-northeastern BOB region and negative anomalies on its two sides (Fig. 1c). Overall, positive centers of rainfall anomalies overlap the heavy climatic rainfall centers along central India-northeastern BOB, and negative centers of rainfall anomalies overlap the heavy climatic rainfalls over the adjacent seas along the west coast of India and the southeastern edge of the TP (Fig. 1c, Supplementary Fig. 1). The anomalous cyclonic circulation is located to the central-east of the climatic IMT (Fig. 1a, Supplementary Fig. 1), signifying a strengthening and eastward extension of the IMT. The SVD1 mode reveals a highly coupled feature of atmospheric circulation and rainfall for the SASM, and the corresponding time series (PC1 hereafter) of SVD1 for 850-hPa wind and rainfall yields a significant correlation coefficient of 0.937 (Fig. 1e).

The second SVD (SVD2 hereafter) mode is responsible for 23.98% of the total squared covariance (Figs. 1b, d, f). The wind field associated with SVD2 displays an anomalous low-level cyclonic circulation over the Indian subcontinent, with westerly anomalies stretching from the Arabian Sea to India south of 20°N and easterly anomalies along the southern edge of the TP (Fig. 1b). Negative anomalies of rainfall appear over the southeastern edge of the TP and positive anomalies over the other parts of South Asia (Fig. 1d). The corresponding time series (PC2 hereafter) of SVD2 for 850-hPa wind and rainfall yield a correlation coefficient of 0.870. Note that the positive center covering the eastern TP, Pakistan, central-northern India, and the interior of the Indochina subcontinent does not well overlap with heavy climatic rainfall (Fig. 1d, Supplementary Fig. 1). Therefore, the overwhelmingly dominant contribution of SVD1 mode in the coupling of the SASM system is proposed, in the concept of its explained covariance and a high degree of coincidence with the climatological features. Broadening or narrowing the domain, or focusing on June-September (JJAS) for the SVD analysis yields almost identical results (Supplementary Figs. 2–4), suggesting the robustness of the two leading modes discussed above.

**Fig. 2:**
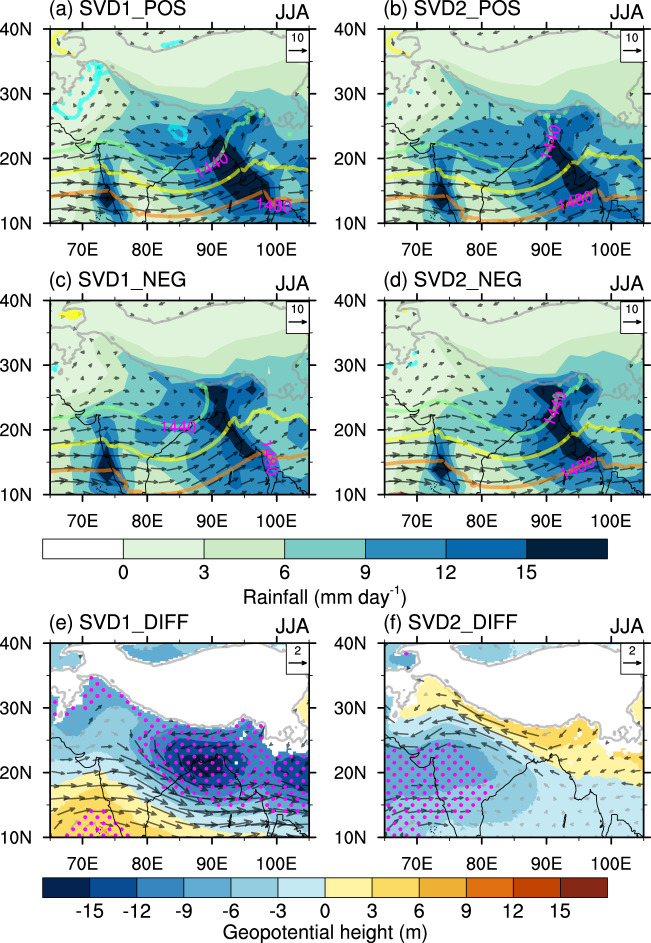
Atmospheric processes associated with the two leading singular value decomposition modes. Composite patterns of rainfall (mm day^−1^; shading), 850-hPa wind (m s^−1^; vector), and 850-hPa geopotential height (m; contour) in **a**, **b** positive and **c**, **d** negative years with respect to the principal components of rainfall. Composite differences (positive minus negative) in 850-hPa wind (m s^−1^; vector) and geopotential height (m; shading) with respect to **e** the first principal component and **f** the second principal component. Significant values exceeding the 95% confidence level are marked by magenta dots or black vectors in **e**, **f**.

To identify the physical processes associated with the two leading modes, we investigate the composite results with respect to PC1 and PC2 (Fig. 2). The positive years are picked when both the PCs of 850-hPa wind and rainfall are above 0.8 standard deviations, and the negative years, below −0.8 standard deviations. The results are not significantly different when the threshold is changed to 1.0 standard deviation (figure not shown). In summer, there is a northwest-southeast elongated belt of low pressure extending from Rajasthan, India to the BOB (Fig. 2a–d), namely, the IMT. Such a low-pressure system brings sufficient rainfall to the central India-northeastern BOB region. Interaction between low-level westerly winds and terrain forms another heavy rainfall zone along the west coast of India and its adjacent seas. During the positive phase of SVD1, the IMT strengthens and extends eastward, with stronger westerlies on its southern flank and anomalous easterlies over region south of the TP (Fig. 2a, c, e). Correspondingly, rainfall increases over central India-northeastern BOB, and decreases over the southeastern edge of the TP possibly due to a weaker moisture transport by the anomalous easterlies. The decreased rainfall over southern India and its adjacent seas is accompanied by increased geopotential height over the region (Fig. 2a, c, e). On the other hand, the IMT is more likely to shift southwestward during the positive phase of SVD2, producing an anomalous cyclonic circulation and above-normal rainfall over the Indian subcontinent (Fig. 2b, d, f). The decreased rainfall over the southeastern edge of the TP is consistent with the weakened southwesterlies (or northeasterly anomalies).

It has long been recognized that ENSO can remotely modulate the SASM, through the inter-seasonal thermal storage in the Indian Ocean, or the simultaneous atmospheric forcing^21,23–28^. We find that the SVD1 mode is significantly correlated with the SSTs in the central-eastern tropical Pacific and the Indian Ocean in the preceding winter and spring seasons, reminiscent of an ENSO decaying process (Supplementary Fig. 5a, c, e). On the other hand, significant SST signals appear over the central-eastern tropical Pacific in the simultaneous summer, but significant signals can hardly be found in the previous seasons for the SVD2 mode (Supplementary Fig. 5b, d, f). The correlation coefficients between PC1 of rainfall and the Niño-3 (Niño-3.4) index in preceding winter and simultaneous summer are −0.581 (−0.542) and 0.199 (0.340), respectively; they are 0.001 (0.021) and −0.267 (−0.343) for the PC2 of rainfall, respectively. Note that the 95% confidence level by the Student-*t* test is 0.304. Similar results are obtained when the PCs of 850-hPa winds are applied. The above analysis suggests that the SVD1 mode exhibits a lagged response to ENSO whereas the SVD2 mode is only connected simultaneously and moderately with SSTs in the central-eastern tropical Pacific, illustrating the superiority in monsoon predictability of the first mode of the SASM.

### Mining an index capable of proxying the predictable dominant mode

Although the PC1 provides a robust index, it is not easily applicable for real-time monitoring and prediction of the SASM. Mining a simple index with a strong physical connection to this predictable dominant mode is much needed.

Table 1 lists nine existing widely used indices for the SASM, which can be dynamically classified into four categories. The first category interprets the north-south thermal contrast, i.e., the Webster-Yang index (WYI)^11^ defined by the vertical shear of zonal winds, and the monsoon Hadley circulation index (hereafter MHI)^12^ defined as the area-averaged meridional wind shear over South Asia. The second category represents the overall convection (rainfall) intensity for South Asia or the Indian subcontinent, including the AIRI^29^, the extended Indian monsoon rainfall index (EIMRI) proposed by Goswami^12^, and the convection index (CI) proposed by Wang and Fan^10^. The third category is a shear vorticity index termed the Indian monsoon index (IMI)^30^. Finally, some indices are based on the seasonality of wind field, i.e., the three SASM indices (briefly denoted as the SASMI, SASMI1, and SASMI2) defined as the area-averaged dynamically normalized seasonality (DNS) within different domains of South Asia^31,32^. Given that the first mode is actually a reflection of the intensity and zonal position of the IMT, which is tightly coupled with rainfall variation, these indices can hardly represent its physical proxy perfectly. A new dynamic index needs to be constructed to fill this gap for monsoon research.

**Table 1:** Description of the South Asian summer monsoon indices

	Define variables, levels (hPa), and regions
WYI^11^	u, 850-200, (40°-110°E, 0°-20°N)
AIRI^29^	Rainfall, all the Indian sub-divisions
EIMRI^12^	Rainfall, (70°-110°E, 10°-30°N,)
MHI^12^	v, 850-200, (70°-110°E, 10°-30°N,)
CI^10^	OLR, (70-100°E, 10°-25°N)
IMI^30^	u, 850, (40°-80°E, 5°-15°N) - (70°-90°E, 20°-30°N)
SASMI^31^	DNS, 850, (35°-97.5°E, 5°-22.5°N)
SASMI1^31^	DNS, 850, (35°-70°E, 2.5°-20°N)
SASMI2^31^	DNS, 850, (70°-110°E, 2.5°-20°N)

The WYI, AIRI, EIMRI, MHI, CI, IMI are abbreviations for the Webster-Yang index, All India Rainfall index, extended Indian monsoon rainfall index, monsoon Hadley circulation index, convection index, and Indian monsoon index. The SASMI, SASMI1, and SASMI2 are the three South Asian summer monsoon indices proposed by Li et al.^31^ u and v represent zonal and meridional wind components, respectively. OLR denotes the out-going longwave radiation. The dynamically normalized seasonality (DNS)^31, 32^ index is given by *σ*_*m,n*_ = ‖${\bar{V}}_{1}$ − *V*_*m,n*_‖ ⁄ ‖$\bar{V}$‖ − 2, where ${\bar{V}}_{1}$, $\bar{V}$, and ${V}_{m,n}$ represent climatological January wind vector, the mean of climatological January and July wind vectors, and the monthly wind vector in the year *n* and month *m*, respectively. For a specific variable A, ‖*A*‖ $=$ (∬_*S*_ |*A*|^2^ *dS*)^1/2^, where S denotes the integration domain.

**Fig. 3:**
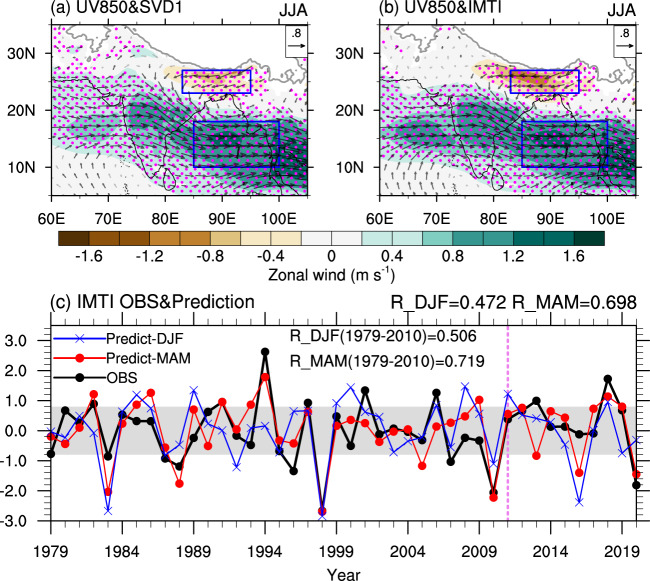
Definition and prediction of the Indian monsoon trough index (IMTI). Regressions of 850-hPa wind (m s^−1^; vector) against **a** the first principal component (PC1) of rainfall and **b** IMTI. **c** Time series of the standardized IMTI in observation (black curve) and that predicted by the sea surface temperature over the eastern tropical Indian Ocean (TEIO_SST) and tropical Atlantic (TA_SST) in preceding spring (red curve) and by Niño-3 index in preceding winter (blue curve). In **a**, **b** shading denotes zonal wind. Significant values exceeding the 95% confidence level are marked by magenta dots or black vectors. The blue boxes outline the regions for the definition of the IMTI. In **c**, “R_DJF” and “R_MAM” represent the correlation coefficients of observed IMTI with inferred IMTI by Niño-3 index and by the TEIO_SST and TA_SST for 1979-2020, respectively. “R_DJF(1979-2010)” and “R_MAM (1979–2010)” represent the correlation coefficients for 1979–2010. The vertical dashed line denotes the line of demarcation between the training period (1979–2010) and the prediction period (2011–2020). Observed values within the gray zone (absolute values ≤ 0.8) represent the normal years; and those below and above the gray zone are for the negative and positive years, respectively.

**Fig. 4:**
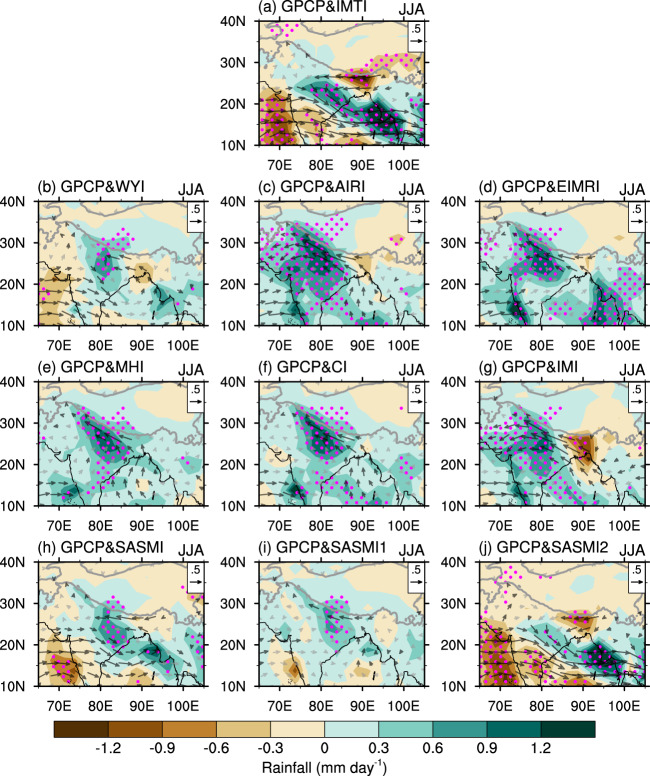
Spatial patterns of 850-hPa wind and rainfall associated with South Asian summer monsoon indices. Regressions of 850-hPa wind (m s^−1^; vector) and rainfall (mm day^−1^; shading) against the **a** Indian monsoon trough index (IMTI), **b** Webster-Yang index (WYI), **c** All India Rainfall index (AIRI), **d** extended Indian monsoon rainfall index (EIMR), **e** monsoon Hadley circulation index (MHI), **f** convection index (CI), **g** Indian monsoon index (IMI), and **h–j** the three South Asian summer monsoon indices (briefly denoted as the SASMI, SASMI1, and SASMI2) proposed by Li et al.^31^. Significant values exceeding the 95% confidence level are marked by magenta dots or black vectors.

Motivated by the physical interpretation of the SVD1 mode, we define a new index using the area-averaged 850-hPa zonal wind over (85°−100°E, 10°−18°N) minus that over (83°−95°E, 23°−27°N). The two domains well overlap with the southern and northern flanks of the low-level cyclonic anomalies associated with the SVD1 (Fig. 1a), as outlined in Fig. 3a, b. Since the basic idea behind this new index is to measure the intensity and zonal position of the IMT, we refer to the index as the IMT index (IMTI). Both the regressions of 850-hPa wind upon the PC1 of rainfall and the IMTI present an anomalous cyclonic circulation from the northern BOB to the south of the TP, and the features are almost identical to each other (Fig. 3a, b). The rainfall anomalies associated with the IMTI also show a northwest-southeast elongated sandwich-like pattern like those associated with the SVD1 (Figs. 1b, 4a). The time series of standardized IMTI, shown in Fig. 3c, exhibits consistent positive and negative years with the PCs of the SVD1 (Fig. 1e). The correlation coefficients between the IMTI and PC1s of 850-hPa wind and rainfall are 0.896 and 0.938, respectively, both exceeding the 99.9% confidence level. Therefore, the IMTI is an appropriate physical proxy for the dominant predictable mode of the SASM.

### Merits of the new index

To objectively evaluate the performance of multiple indices in measuring the SASM, correlations between each index and each of the two leading modes are examined (Table 2). Most of the existing indices (i.e., the AIRI, EIMR, MHI, CI, and IMI) are significantly correlated with the SVD2 mode, instead of the SVD1 mode, while both the SASMI and SASMI2 exhibit reverse relationship with the two modes (Table 2). The other two indices (i.e., the WYI and SASMI1), however, are not well correlated with either SVD1 or SVD2. This is because the WYI primarily measures a broad-scale SASM rather than the regional features and the SASMI1 targets Southwest Asia, which is located west of the study domain.

**Table 2:** Correlation coefficients between the South Asian summer monsoon indices and principal components (PCs), and the preceding winter and simultaneous summer Niño-3 indices

	PC1	PC2	Niño3_DJF	Niño3_JJA
IMTI	0.896**	0.246	−0.472**	0.190
WYI	0.268	0.148	−0.330*	−0.632**
AIRI	−0.136	0.790**	0.057	−0.442**
EIMR	0.063	0.616**	−0.001	−0.253
MHI	−0.129	0.373*	−0.012	−0.392*
CI	−0.025	0.480**	0.025	−0.491**
IMI	0.099	0.686**	0.103	−0.486**
SASMI	0.439**	0.177	−0.335*	−0.235
SASMI1	0.059	0.158	−0.095	−0.336*
SASMI2	0.878**	−0.070	−0.476**	0.119

Column 1 lists the multiple South Asian summer monsoon indices, and columns 2-5 present correlation coefficients for the first and second PCs of rainfall, and the Niño-3 index in the preceding winter (Niño3_DJF) and simultaneous summer (Niño3_JJA), respectively. The WYI, AIRI, EIMRI, MHI, CI, IMI are abbreviations for the Webster-Yang index, All India Rainfall index, extended Indian monsoon rainfall index, monsoon Hadley circulation index, convection index, and Indian monsoon index. The SASMI, SASMI1, and SASMI2 are the three South Asian summer monsoon indices proposed by Li et al.^31^. Endings with one/two asterisks denote the values exceeding the 95%/99% confidence level.

Figure 4 shows the relationships of the indices with rainfall and atmospheric circulation anomalies over South Asia. The regression patterns associated with the AIRI, EIMR, MHI, CI, and IMI present positive anomalies of rainfall over most parts of South Asia and low-level cyclonic circulation anomalies over India, similar to those for the SVD2 mode. Nevertheless, none of these indices reflect rainfall anomalies over the southeastern edge of the TP, with the exception of the IMI. Rainfall and circulation anomalies associated with the SASMI and SASMI2 are similar to those associated with the SVD1 mode. Compared with the IMTI, the SASMI reflects overall weaker rainfall and circulation anomalies, and the SASMI2 is associated with weaker rainfall anomalies over central India and the southeastern edge of the TP. The anomalous pattern associated with the WYI is similar to that with the SVD1 mode, displaying a quite weaker magnitude. On the other hand, significant anomalies only appear over the northeastern corner of India for the SASMI1. Rainfall anomalies derived from the Global Precipitation Climatology Centre (GPCC) and Asian Precipitation-Highly Resolved Observational Data Integration toward Evaluation of Water Resources (APHRODITE) replicate the patterns similar to those from the Global Precipitation Climatology Project (GPCP) (Supplementary Figs. 6, 7).

Relationships of each index with the SST signals in preceding and simultaneous seasons are examined next. Consistently, the indices that are closely connected with the SVD1 mode (i.e., IMTI, SASMI, and SASMI2) are significantly related to the Niño-3 index in the preceding winter, whereas those associated with the SVD2 mode (i.e., AIRI, CI, MHI, and IMI) are more likely correlated with the Niño-3 index in simultaneous summer significantly (Table 2). In particular, the correlation coefficients of the preceding Niño-3 index with the IMTI and SASMI2 are statistically significant at the 99.9% confidence level. For the 8 positive years (1982, 1991, 1994, 1997, 2001, 2006, 2013, and 2018) and 8 negative years (1983, 1987, 1988, 1996, 1998, 2007, 2010, and 2020) of IMTI (see Fig. 3c), there are 4 positive years occurred during the decaying process of La Niña (1997, 2001, 2006, and 2018) and 7 negative years occurred during the decaying process of El Niño (1983, 1987, 1988, 1998, 2007, 2010, and 2020). ENSO years are picked according to the criterion of Climate Prediction Center for (https://origin.cpc.ncep.noaa.gov/products/analysis_monitoring/ensostuff/ONI_v5.php). That is, 11 out of 16 abnormal IMTI years are corresponded well to ENSO decaying process. For the five slip-through-the-nets, there are 4 normal years of ENSO (1982, 1991, 1994, and 2013), and only 1996 is La Niña decaying year which is expected to be El Niño decaying year according to the negative correlation between the IMTI and ENSO index. The WYI is correlated with the simultaneous Niño-3 index highly (−0.632) but with the preceding winter Niño-3 index moderately (−0.330), and the SASMI1 shows a moderately significant correlation with the simultaneous Niño-3 index. These results suggest that the IMTI, SASMI, SASMI2, and WYI have a higher predictability than the others, in particular for the IMTI and SASMI2. Similar results can be obtained when the Niño-3.4 index is used instead.

**Fig. 5:**
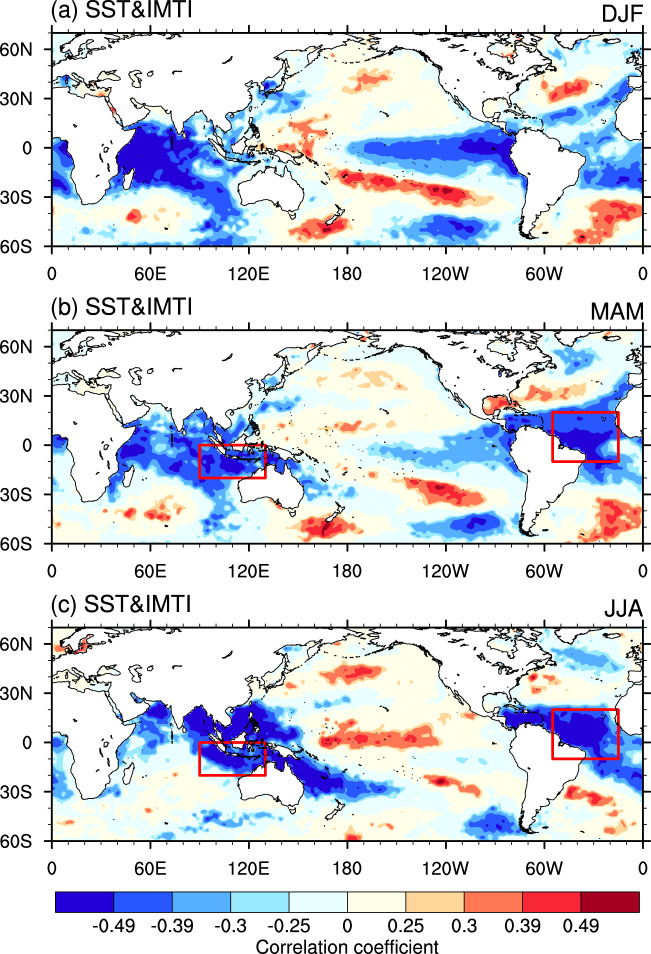
Indian monsoon trough index (IMTI)-predictor correlations. Correlation of sea surface temperature in **a** preceding winter, **b** preceding spring, and **c** simultaneous summer with the IMTI. The absolute values above 0.25, 0.30, 0.39, and 0.49 indicate that they significantly exceed the 90%, 95%, 99%, and 99.9% confidence levels, respectively. The red boxes outline the regions for the predictors.

We thus conclude that the new index has several obvious merits: (1) a strong physical connection and identical representation of the most dominant mode of the SASM, (2) its simplicity for real-time monitoring, and (3) high predictability. Here, a statistical prediction of the IMTI is provided based on the physical linkage between the index and the preceding SST signals. As shown in Fig. 5, the correlation pattern between the IMTI and the SST from preceding winter to simultaneous summer exhibits a mature-decaying process of ENSO, bearing a large resemblance to that for the SVD1 mode (Supplementary Fig. 5). The SSTs in the central-eastern tropical Pacific, tropical Indian Ocean, and tropical Atlantic show high correlations with the IMTI. While the SST signals in the central-eastern tropical Pacific attenuate rapidly after winter season, those in the tropical Indian Ocean and tropical Atlantic persist across the three seasons. We select two predictors in preceding spring for one-season lead prediction: the area-averaged SSTs over the eastern tropical Indian Ocean (90°-130°E, 0°-20°N) and tropical Atlantic (305°-345°E, 10°S-20°N), referred to as the TEIO_SST and TA_SST, respectively. Both the TEIO_SST and TA_SST persist well from spring to summer (see Fig. 5b, c), and are highly correlated with the IMTI (−0.567 and −0.597, respectively). The two predictors are also significantly correlated with the preceding winter ENSO, with correlation coefficients exceeding the 99.9% confidence level (0.681 and 0.399, respectively). On the other hand, only a moderate correlation is found between the two predictors (0.372). Afterwards, the least absolute shrinkage and selection operator (LASSO) is adopted to construct a regression function with the two predictors. The equation derived from the training period of 1979–2010 is IMTI = −3.174 × TEIO_SST-2.202 ×  TA_SST + 1627.092. The overall skill is quite satisfactory for the entire 42 years (1979–2020), which reaches a significant correlation coefficient of 0.698 (exceeding the 99.9% confidence level) between the observed and inferred IMTI (Fig. 3c). Specifically, the correlation coefficient between the observed and inferred IMTI is 0.719 for the training period, and there are only two false hits (years 2013 and 2016) during the validation period of 2011–2020 (Fig. 3c). In fact, the prediction skill is also significant even if only the preceding winter Niño-3 index (Niño-3_DJF) is employed to predict the IMTI. The equation derived from the training period of 1979–2010 is IMTI = −0.841 $\times$ Niño-3_DJF + 260.0168. The correlation coefficient between the observed IMTI and that inferred by Niño-3 index is 0.472 for the entire period and 0.506 for the training period, respectively, both exceeding the 99% confidence level.

## Discussion

The two most dominant modes of the interannual variability of the SASM, which account for the majority of the total squared covariance, have clear implications on both rainfall intensity and atmospheric circulation anomalies. The first mode is physically linked to the intensity and zonal extension (or contraction) of the IMT, which is coupled with rainfall anomalies over the hardest hit area in terms of climatology (i.e., central India-northeastern BOB regions and the southeastern edge of the TP). The second mode is associated with a meridional shift of the IMT, which is coupled with the opposite anomalies of rainfall over the southeastern edge of the TP and the rest of South Asia. The first mode is highly robust and captures more than half of the total squared covariance, and thus is recommended to be an objective measure of the SASM variation. Importantly, this mode is highly predictable, while the second mode shows a low predictability.

With elaborations on the dynamic features of different modes, it is not surprising to see disparate measures of the SASM strength given by different indices (e.g., the WYI and AIRI)^33,34^. An explanation for this discrepancy is that the indices are respectively connected to different modes of monsoon variation. Accordingly, the multiple SASM indices can be basically re-classified into two groups: the first mode-related including the IMTI, WYI, SASMI, and SASMI2, and the second mode-related including the AIRI, EIMR, MHI, CI, and IMI. The SASMI1, however, is a reflection of the monsoon features over west of India rather than the core region of South Asia, and thus is excluded from both groups. Most of the existing indices cannot reflect rainfall anomalies over central India or the southeastern edge of the TP. Although the SASMI2 shows a comparable correlation with the first mode and the previous tropical SST anomalies as the new index (the IMTI), it has a different physical meaning from the first mode. On the other hand, the simple new index is connected to the same dynamic system (namely, the IMT) and represents the first mode with a high fidelity, showing advantages of representativeness and objectiveness of the coupling SASM system.

Most of the existing indices tend to be associated with the second mode (such as the AIRI, EIMR, MHI, CI, and IMI) or moderately correlated with the first mode (such as the WYI and SASMI), which seem to illustrate that the SASM is unpredictable. We point out this is not true by providing compelling evidence in this paper. The new index defined according to the first mode is well predicted by a statistical model one-season ahead, with a skill of *R* = 0.698 for the recent 42 years, suggesting that at least 48.7% of the SASM variance is predictable. The statistical equation is constructed solely based on the physical connection between the new index and previous SST anomalies, and we are working hard to reach a higher prediction skill in the near future. It should be pointed out that, among the existing indices, the SASMI2 is highly associated with the first mode and the preceding winter ENSO, suggesting that the SASMI2 may share some common physical characteristics with the IMTI, which needs to be further explored.

The present study points out the excellent predictable prospect of the SASM by highlighting the role of the first mode, different from the conventional wisdom. Here, we provide a further physical understanding of the superior predictability of the first mode than the second mode. The lack of significant oceanic signals in the previous seasons contributes to limited predictability of the latter. On the other hand, the former is closely linked to persistent SST anomalies from spring to summer during the decaying phase of ENSO. It has been well recognized that the tropical Indian Ocean was characterized by basin-wide warming during El Niño decaying summer^22^. As reported in previous studies, there are at least two ways by which the Indian Ocean warming can affect the SASM^35–37^. On one hand, warm SST in the southeastern tropical Indian Ocean can strengthen the local Hadley circulation, and thus suppress convection over the northern BOB and central India; the resultant local diabatic cooling can excite a local low-level anticyclone and in turn weaken the IMT and hence the SASM^35,38,39^. On the other hand, both the Indian Ocean warming and the cooling in the western North Pacific during El Niño decaying summer can suppress convection over the western North Pacific^22,40^. And the resultant diabatic cooling can excite westward propagating atmospheric cold Rossby wave, inducing an anomalous anticyclone over South Asia and thus weakening the SASM^35–37^. In addition, the first mode is more closely associated with atmospheric anomalies at the lower latitudes than the second mode (Fig. 1), and is thus more susceptible to the tropical forcing. Moreover, given that large anomalies associated with the first mode appear on the eastern part of South Asia, the TP blocks the influences from the mid-to-high latitudes for the first mode. The second mode, however, is closely linked to the atmospheric forcing at the mid-to-high latitudes and determined largely by the internal atmospheric variability^41,42^ (i.e., the wave train propagating along the westerly jet stream). Another possible reason is that the year-to-year variability of the first mode may be largely due to sampling fluctuations associated with the intra-seasonal variability, regarding to its tight connection with the IMT^21,43,44^. The 10-20-day and 30-60-day oscillations will introduce considerable tropical forcing for the first mode of interannual variability. This raises another merit of the new index, that is, measuring the SASM on various time scales. As an example, the time series of the standardized 5-day running averaged IMTI in 1994 is shown (Supplementary Fig. 8), which exhibits apparent intra-seasonal oscillation. Patterns of rainfall and circulation anomalies similar to those associated with the first mode are obtained from the composite differences between the active and inactive periods (Supplementary Fig. 8). The multi-scale real-time monitoring and prediction of using the new index can be anticipated.

## Methods

### Data sets

The monthly data sets of 850-hPa wind and geopotential height are obtained from the European Centre for Medium-range Weather Forecasts Reanalysis v5^45^ (ERA5), which have a high resolution of 0.25° × 0.25°. The monthly SST with a resolution of 1°×1° is from the Hadley Centre Sea Ice and Sea Surface Temperature data set (HadISST) Version 1.1^46^. The monthly rainfall from the GPCP Version 2.3 with a resolution of 2.5° × 2.5° is utilized in this study^47^. The monthly rainfall from the GPCC^48,49^ Version 2020 with a resolution of 1°×1° and the daily rainfall from the APHRODITE V1101 and V1101EX_R1 are used for validation^50^. All the data sets cover the period of 1979–2020, except for the APHRODITE with a shorter period of 1979–2015. The AIRI for 1979–2019 is obtained from the National Informatics Centre, Ministry of Electronics and Information Technology, Government of India. The SASMI, SASMI1, and SASMI2 for 1979–2020 are obtained from the author’s website.

### SVD analysis

To identify the major coupled features of atmospheric circulation and rainfall during the SASM, SVD analysis was performed on 850-hPa wind and rainfall over South Asia. The SVD isolates the dominant modes of cross-covariance between the data sets. The output consists of the left matrix and right matrix, and each mode has a unique eigenvalue representing the percentage of squared covariance explained by the specific mode, which decreases with the order of the modes. The corresponding temporal variations (i.e., the PCs) for the two matrices are similar to each other with high correlation coefficients, signifying their highly coupled features.

### Correlation and significance test

Here, correlation coefficient r is calculated as follows:

1 $${{{{{\rm{r}}}}}}=\frac{\mathop{\sum }\nolimits_{i=1}^{n}({x}_{i}-\bar{x})({y}_{i}-\bar{y})}{\sqrt{\mathop{\sum }\nolimits_{i=1}^{n}{({x}_{i}-\bar{x})}^{2}}\sqrt{\mathop{\sum }\nolimits_{i=1}^{n}{({y}_{i}-\bar{y})}^{2}}}$$

where $\bar{x}$ and $\bar{y}$ represent the mean values of variables ${x}_{i}$ and ${y}_{i}$ from *i* = 1 to *i* = *n* (*n* is the sample size).

The statistical significance level is calculated based on the Student’s *t*-test, using the equation:

2 $$t=\frac{\bar{x}-\bar{y}}{\sqrt{\frac{\left(m-1\right){s}_{1}^{2}+\left(n-1\right){s}_{2}^{2}}{m+n-2}}\sqrt{\frac{1}{m}+\frac{1}{n}}}$$

where m and n are the sample sizes for variables ${x}_{i}$ and ${y}_{i}$, respectively. $\bar{x}$ and $\bar{y}$ are the mean values of these variables. ${s}_{1}^{2}$ and ${s}_{2}^{2}$ are the variances of ${x}_{i}$ and ${y}_{i}$, while $m+n-2$ is the degree of freedom.

In this study, the values of correlation coefficient (r) above 0.304, 0.393, and 0.490 are used to estimate the 95%, 99%, and 99.9% confidence levels, respectively, for a 42-year record length of 1979–2020, according to the Student’s *t*-test. The values should be 0.308, 0.398, and 0.495 for the AIRI correlation with the 41-year record length of 1979–2019.

### LASSO

The LASSO is one of the extensively used linear regression analysis method, with a regularization term expressed as the L_1_ norm of the coefficients in order to enhance the interpretability and prediction skill^51,52^. In this study, the training period and prediction period are 1979–2010 and 2011–2020, respectively. The 5-fold cross-validation is performed in the training period to optimize the lambda value in the regularization term of LASSO regression equation. The 5-fold cross-validation means that the training datasets were split into 5 groups, and the fitting was performed 5 times. For each time, it takes one group as a test dataset, and the remaining 4 groups as a training dataset. The explained variances of the training period and prediction period are 51.66% and 34.37%, respectively. The root mean square errors of the two periods are 1.19 and 1.24, respectively.

## Supplementary information

Supplementary Information

## Data Availability

The ERA5 is publicly available at https://www.ecmwf.int/en/forecasts/datasets/reanalysis-datasets/era5. The HadISST is publicly available at https://www.metoffice.gov.uk/hadobs/hadisst. The GPCP and GPCC are publicly available at https://psl.noaa.gov/data/gridded/data.gpcp.html and https://www.dwd.de/EN/ourservices/gpcc/gpcc.html, respectively. The APHRODITE is publicly available at http://aphrodite.st.hirosaki-u.ac.jp/products.html. The AIRI is obtained from https://data.gov.in/catalog/rainfall-india, and the SASMI, SASMI1, and SASMI2 are publicly available at http://lijianping.cn/dct/page/65576.

## Code availability

All figures in this article are produced by the NCAR Command Language (NCL) version 6.6.2, and the source codes can be obtained upon request to the corresponding author. The LASSO model can be obtained from the Scikit-learn version 0.24.2 (https://scikit-learn.org/stable/modules/generated/sklearn.linear_model.LassoCV.html).
