# Author Correction: A predictable prospect of the South Asian summer monsoon

**DOI:** 10.1038/s41467-023-36831-3

**Published:** 2023-03-14

**Authors:** Tuantuan Zhang, Xingwen Jiang, Song Yang, Junwen Chen, Zhenning Li

**Affiliations:** 1 School of Atmospheric Sciences, Sun Yat-sen University, Southern Laboratory of Ocean Science and Engineering (Zhuhai), Zhuhai, Guangdong 519082 China; 2 Guangdong Province Key Laboratory for Climate Change and Natural Disaster Studies, Sun Yat-sen University, Zhuhai, Guangdong 519082 China; 3 Heavy Rain and Drought-Flood Disasters in Plateau and Basin Key Laboratory of Sichuan Province, Institute of Plateau Meteorology, China Meteorological Administration, Chengdu, Sichuan 610072 China; 4 Shenzhen Wiselec Technology Co., Ltd., Shenzhen, Guangdong 518048 China; 5 Division of Environment and Sustainability, The Hong Kong University of Science and Technology, Hong Kong, China

**Keywords:** Atmospheric dynamics, Projection and prediction

Correction to: *Nature Communications* 10.1038/s41467-022-34881-7, published online 18 November 2022

In this article the affiliation details for Xingwen Jiang were incorrectly given as ‘Plateau Atmosphere and Environment Key Laboratory of Sichuan Province, Institute of Plateau Meteorology, China Meteorological Administration, Chengdu, Sichuan, 610072, China’ but should have been ‘Heavy Rain and Drought-Flood Disasters in Plateau and Basin Key Laboratory of Sichuan Province, Institute of Plateau Meteorology, China Meteorological Administration, Chengdu, Sichuan, 610072, China’.

The original article has been corrected.

