# Supplementary material for: A predictable prospect of the South Asian summer monsoon
Source: Nat Commun. 2022 Nov 18;13:7080. doi: 10.1038/s41467-022-34881-7 (PMC9674705; doi:10.1038/s41467-022-34881-7)
Supplement: Supplementary file 1 — Supplementary Information [file 41467_2022_34881_MOESM1_ESM.pdf]

Supplementary Information for

A Predictable Prospect of the South Asian Summer Monsoon

Tuantuan Zhang[1,2], Xingwen Jiang[3]*, Song Yang[1,2], Junwen Chen[4], and Zhenning Li[5]

[1] School of Atmospheric Sciences, Sun Yat-sen University, Southern Laboratory of Ocean Science and Engineering (Zhuhai), Zhuhai, Guangdong 519082, China

[2] Guangdong Province Key Laboratory for Climate Change and Natural Disaster Studies, Sun Yat-sen University, Zhuhai, Guangdong 519082, China

[3] Plateau Atmosphere and Environment Key Laboratory of Sichuan Province, Institute of Plateau Meteorology, China Meteorological Administration, Chengdu, Sichuan 610072, China

[4] Shenzhen Wiselec Technology Co., Ltd., Shenzhen, Guangdong 518048, China

[5] Division of Environment and Sustainability, The Hong Kong University of Science and Technology, Hong Kong, China

*To whom correspondence should be addressed. xingwen.jiang@yahoo.com.

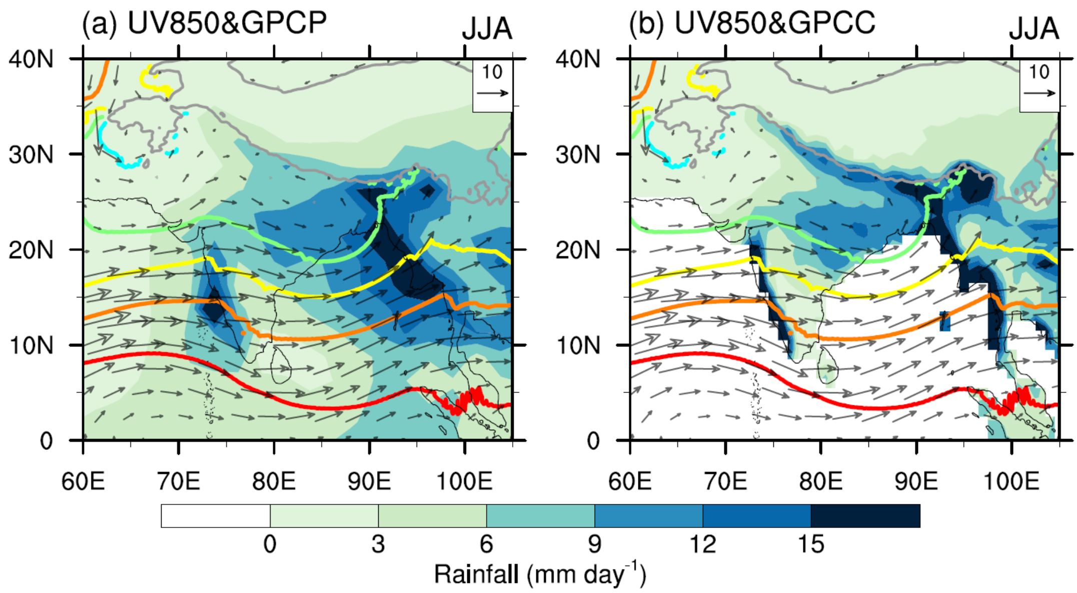

Supplementary Figure 1 | Climatological features of atmospheric condition over South Asia in summer (June-July-August). (a-b) Climatological rainfall (mm day^{-1}; shading), 850-hPa winds (m s^{-1}; vectors), and 850-hPa geopotential height (m; contour) during 1979-2020. Monthly data of rainfall is derived from (**a**) Global Precipitation Climatology Project and (**b**) Global Precipitation Climatology Centre, respectively. Winds and geopotential height are from European Centre for Medium-range Weather Forecasts Reanalysis v5. The red, orange, yellow, and palegreen contours represent geopotatial height of 1500, 1480, 1460, and 1440 m, respectively.

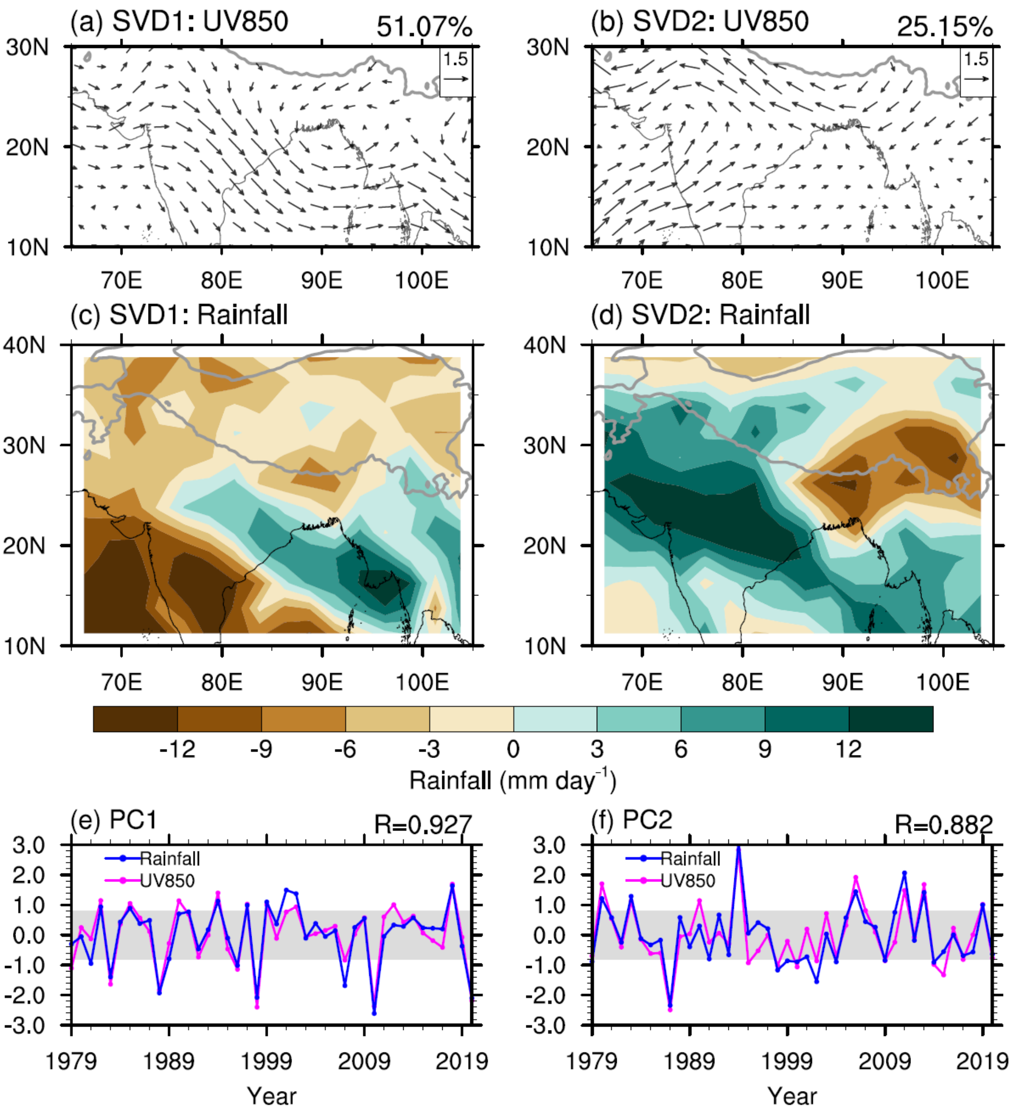

Supplementary Figure 2 | The first and second singular value decomposition modes of South Asian summer monsoon. Same as in Figure 1 in the main text, but the singular value decomposition modes are calculated for June to September.

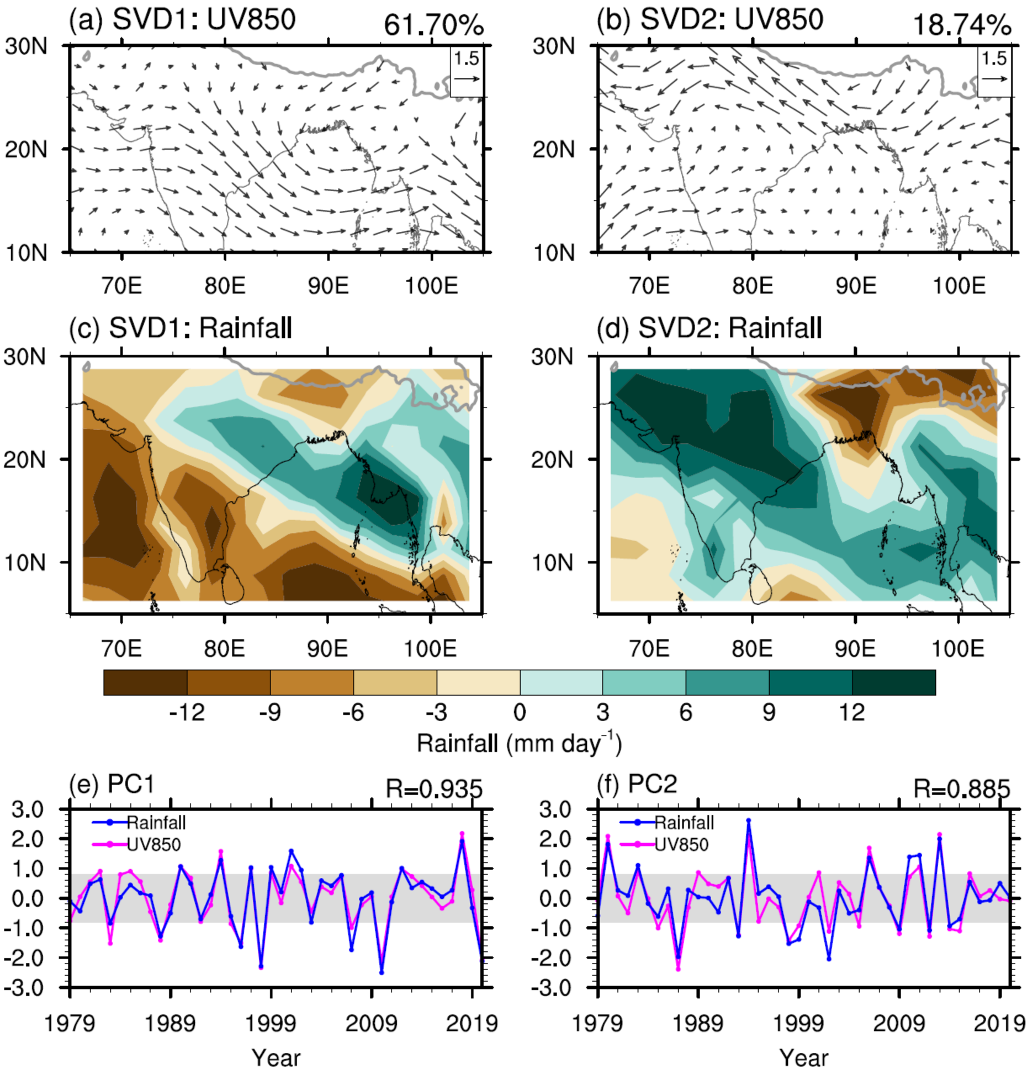

Supplementary Figure 3 | The first and second singular value decomposition modes of South Asian summer monsoon. Same as in Figure 1 in the main text, but for a smaller domain of South Asia.

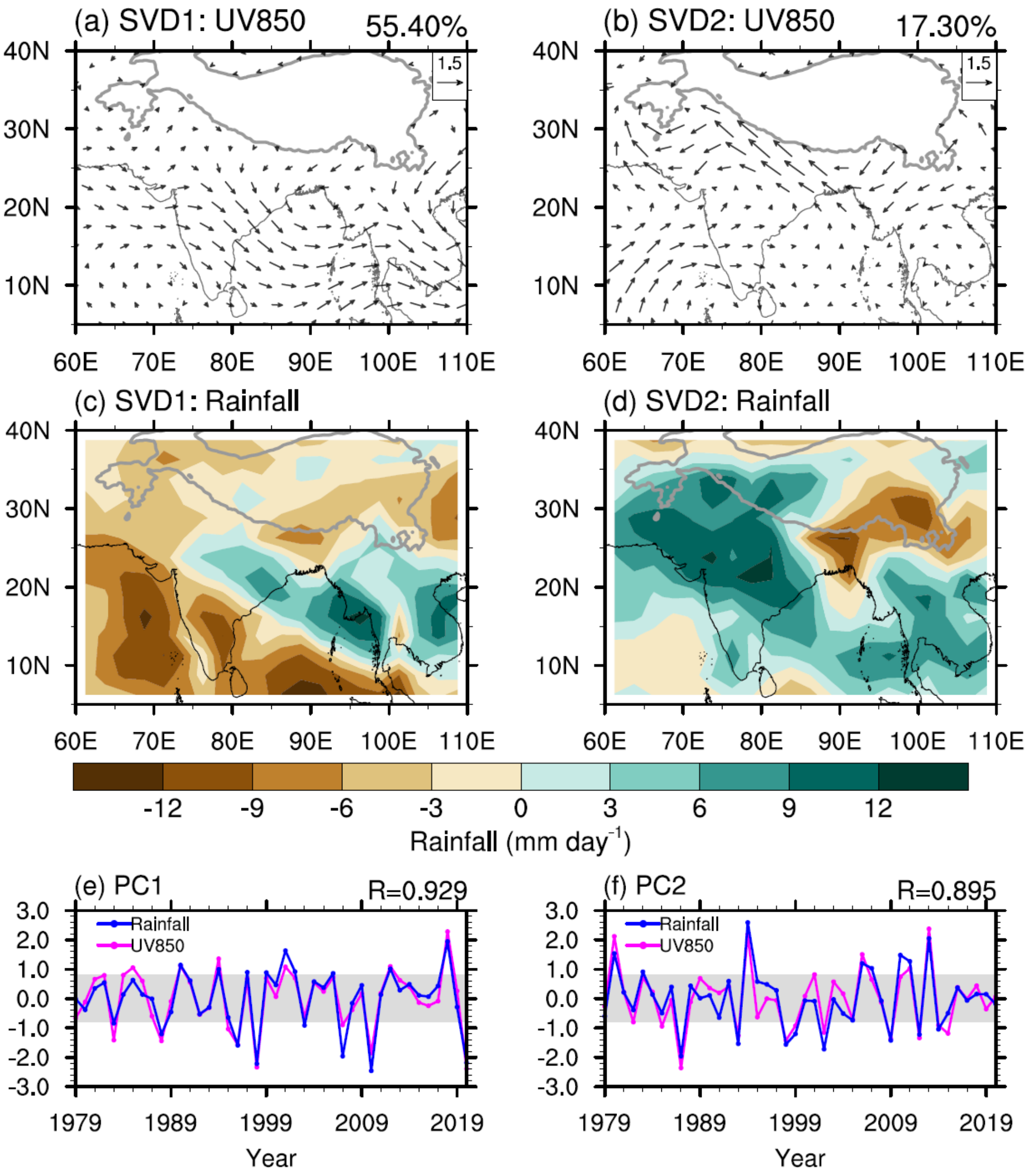

Supplementary Figure 4 | The first and second singular value decomposition modes of South Asian summer monsoon. Same as in Figure 1 in the main text, but for a larger domain of South Asia.

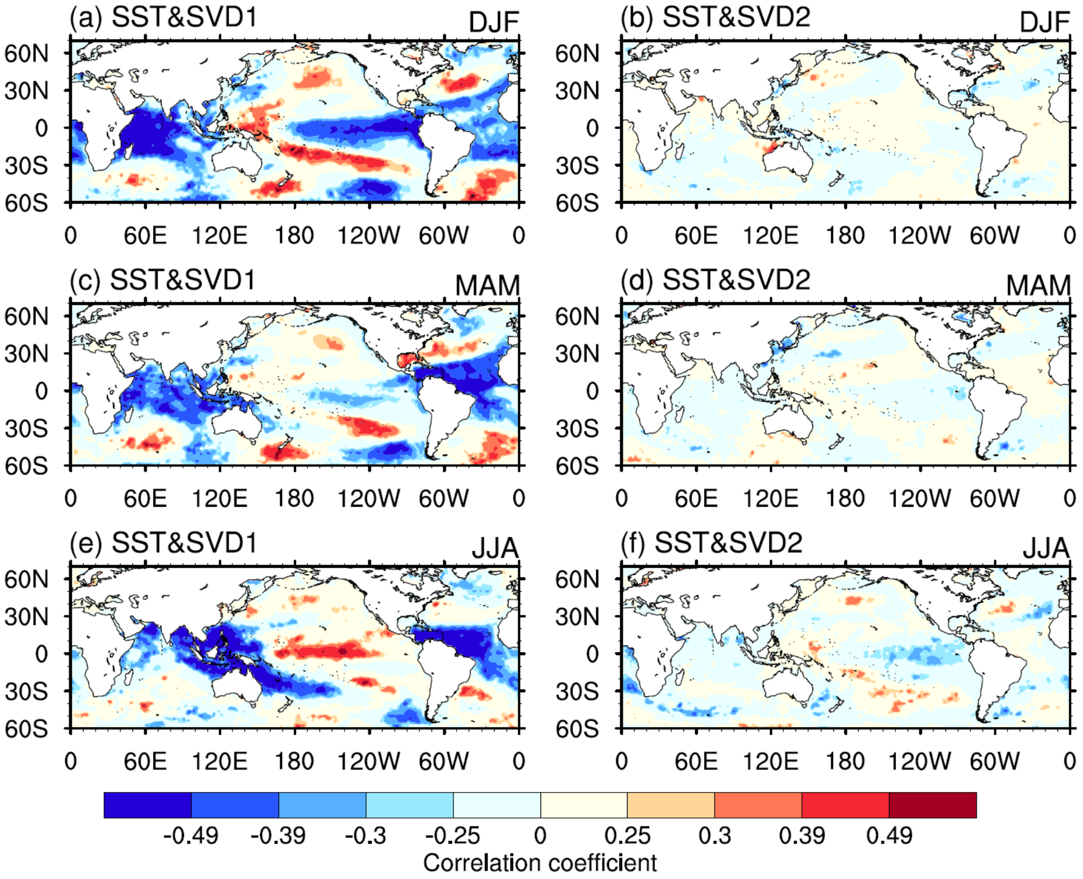

Supplementary Figure 5 | Precursors for the first and second singular value decomposition modes. Correlation of sea surface temperature in (**a-b**) preceding winter, (**c-d**) preceding spring, and (**e-f**) simultaneous summer with the first principal component (PC1) (**left**) and the second principal component (PC2) (**right**) of rainfall. Absolute values above 0.25, 0.30, 0.39, and 0.49 indicate that they significantly exceed the 90%, 95%, 99%, and 99.9% confidence levels, respectively.

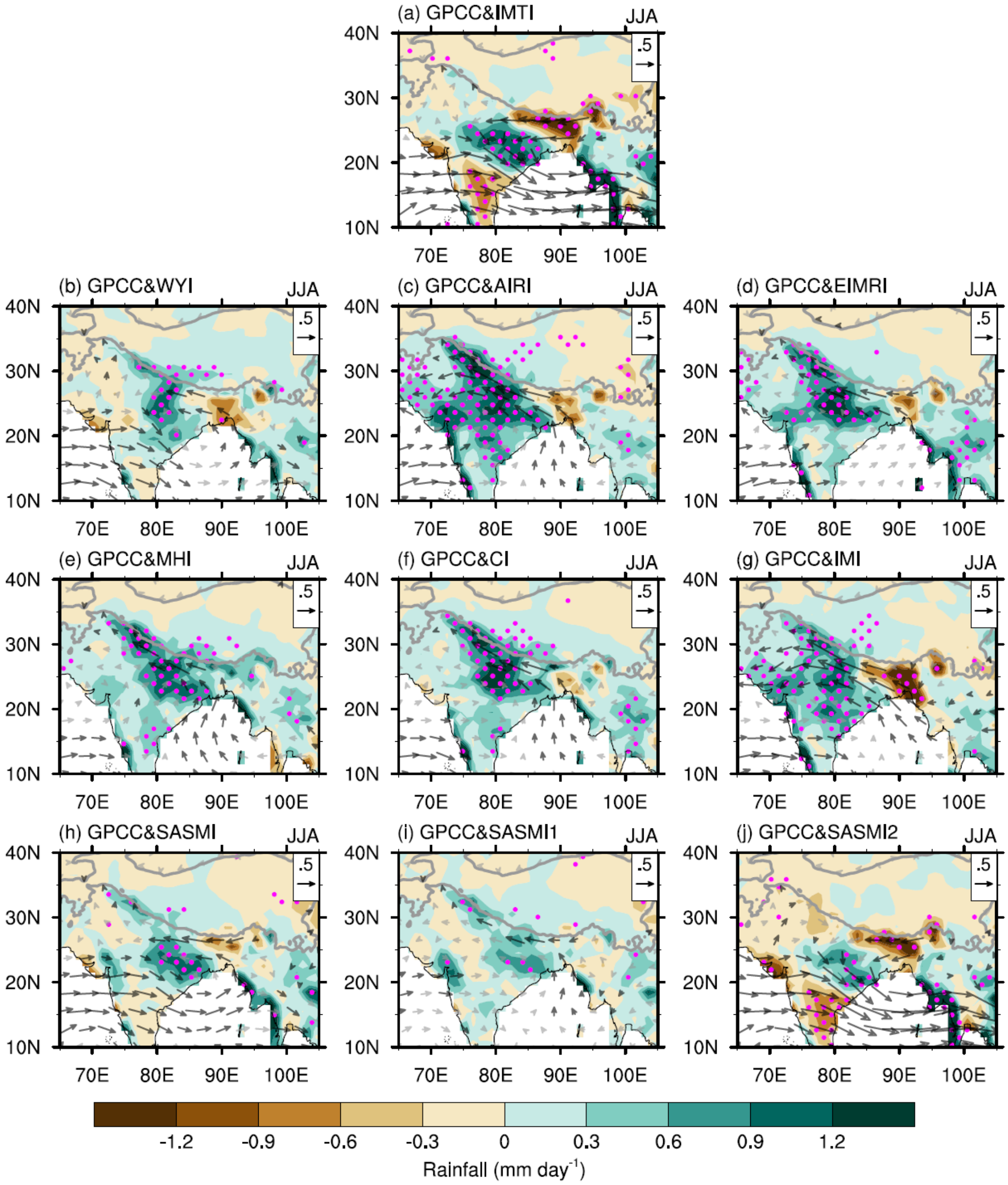

Supplementary Figure 6 | Spatial patterns of 850-hPa winds and rainfall associated with the South Asian summer monsoon indices. Same as in Figure 4 in the main text, but rainfall is derived from Global Precipitation Climatology Centre.

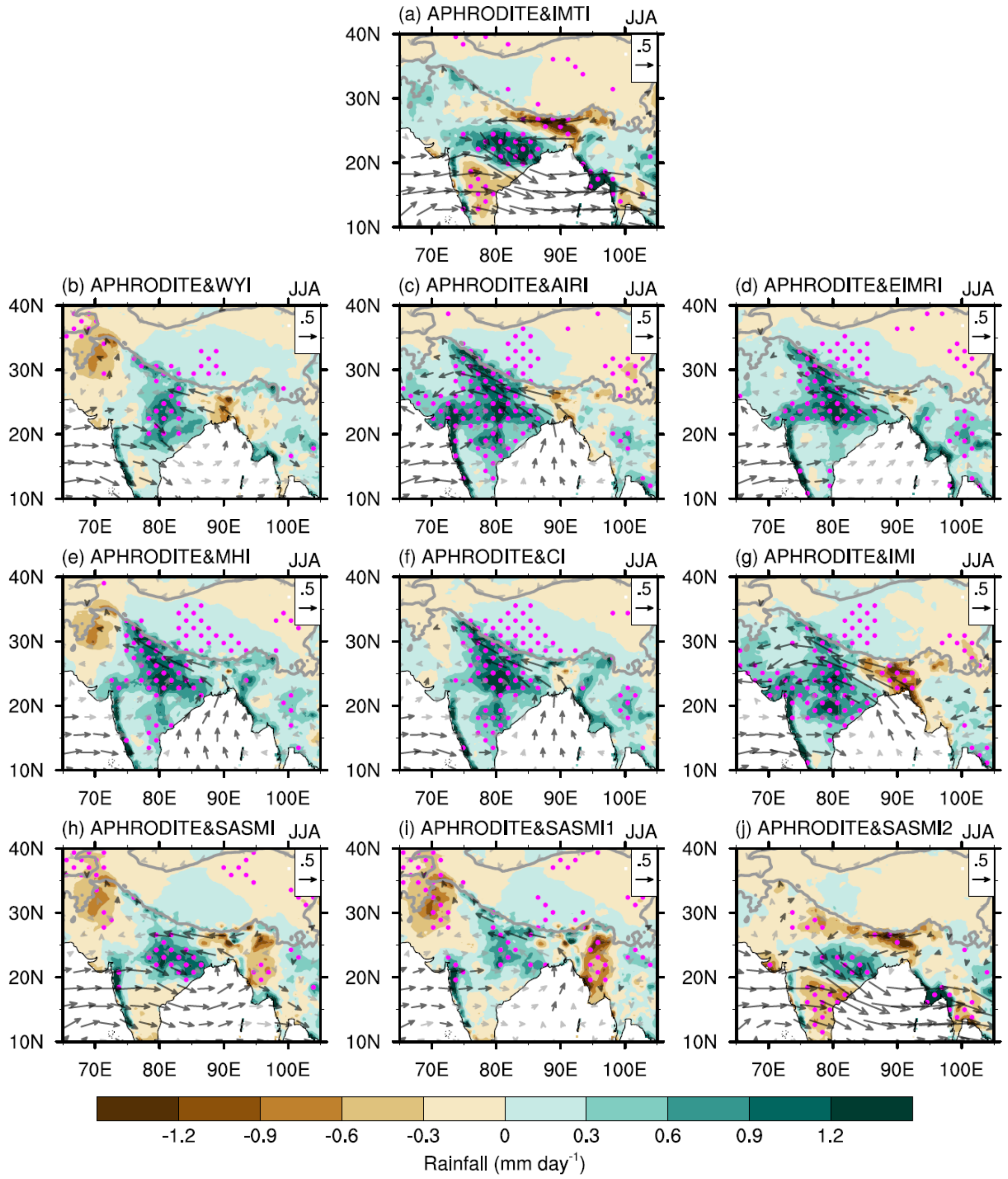

Supplementary Figure 7 | Spatial patterns of 850-hPa winds and rainfall associated with the South Asian summer monsoon indices. Same as in Figure 4 in the main text, but rainfall is derived from Asian Precipitation-Highly Resolved Observational Data Integration toward Evaluation of Water Resources for the period of 1979-2015.

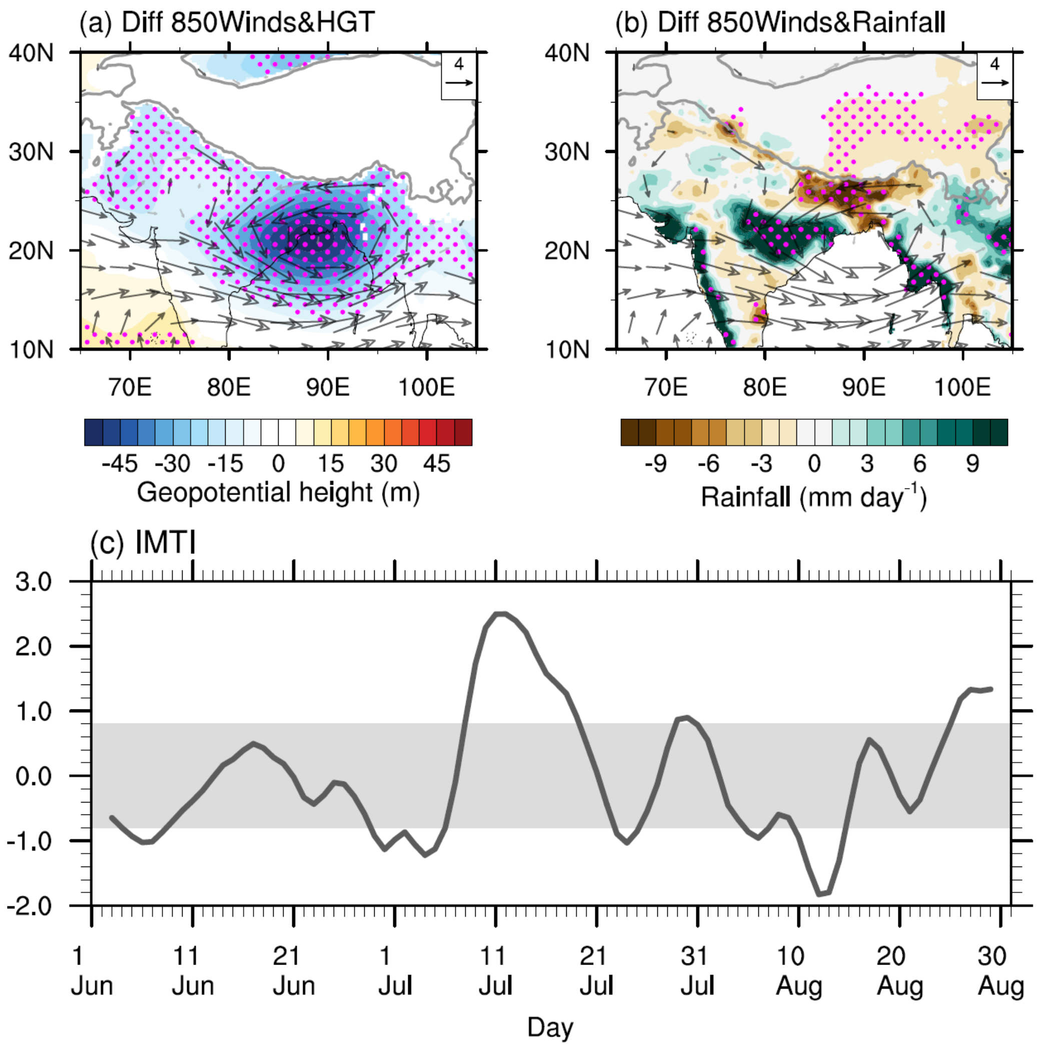

Supplementary Figure 8 | Sub-seasonal atmospheric processes associated with the Indian monsoon trough index (IMTI). Composite differences in (**a**) 850-hPa winds (m s^{-1}; vectors) and geopotential height (m; shading), and (**b**) 850-hPa winds (m s^{-1}; vectors) and rainfall (mm day^{-1}; shading) between the active and inactive periods with respect to the IMTI in 1994. (**c**) Time series of the standardized 5-day running averaged IMTI in 1994. The active (inactive) period is defined as the standardized 5-day running averaged IMTI consecutively exceeding 0.8 (bellowing -0.8) standard deviations for 5 days, which is outside the gray zone. The daily 850-hPa winds and geopotential height are derived from European Centre for Medium-range Weather Forecasts Reanalysis v5,

and the daily rainfall is from Asian Precipitation-Highly Resolved Observational Data Integration toward Evaluation of Water Resources, respectively.
